# Supplementary material for: Cohort-profile: Household transmission of SARS-CoV-2 in a low-resource community in Rio de Janeiro, Brazil
Source: BMJ Open. 2022 Dec 7;12(12):e067212. doi: 10.1136/bmjopen-2022-067212 (PMC9729844; doi:10.1136/bmjopen-2022-067212)
Supplement: Supplementary data [file bmjopen-2022-067212supp001.pdf]

Supplementary Material

Informed Consent Form

Household Transmission and Immunity to SARS-CoV-2 among Paediatric Clients of a Primary Care Center in a Low-resource Community in Rio de Janeiro.

You are being invited to participate in a study that will investigate the coronavirus, hereafter SARS-CoV-2, among people without symptoms because you may have been exposed to the virus or because your SARS-CoV-2 test was positive.

Since December 2019, SARS-CoV-2 has spread rapidly in China and around the world. As of November 2020, 47 million cases have been reported in 189 countries. During this period, a total of 5.6 million cases were reported in Brazil.

The aim of this study is to investigate the clinical progression of SARS-CoV-2 in persons with and without symptoms. In connection with this, the study will assess whether your body is producing antibodies and other substances in response to the virus. We will also study whether people who live or work in your home become infected.

If you agree to participate in the study we will complete a form with information about your symptoms, related medical conditions, and any medications that you take. With your permission, we will collect 4 mL of your blood, which is approximately the same volume as a soup spoon. The blood will be collected via a venal puncture with a disposable needle by a nurse or a trained lab technician. The blood sample will be used to carry out lab exams to detect antibodies or markers of inflammation caused by SARS-CoV-2 infection. We will also collect nasal and throat secretions using three long, thin, and sterile cotton swabs (Q-tips). A swab will be placed in your mouth and

moved in the direction of your tonsils. Another swab will be placed in each nostril and moved in the direction of your nasopharynx, which is approximately 6-8 cm deep within the nasal cavity. We will also collect a fecal sample via a Q-tip inserted into your anus to a depth of approximately 1 cm. You can collect the fecal sample yourself following instructions that we will give you. We will search for SARS-CoV-2 in these samples and attempt to determine its characteristics.

If your test for the coronavirus is negative, we will stay in touch with you to follow the progression of your symptoms. If your test is positive, you will be accompanied by the study for about six weeks. This will involve doing the exams mentioned above about once a week, for a total of 3-4 sample collections. The purpose of the exams will be to see for how long you will have the virus and to detect the appearance of antibodies. If you need to be hospitalized, these tests will be done every 48 hours, on the day when you are released, and six weeks later. You can participate in this study via phone/WhatsApp, social media (Facebook), video calls, or home visits.

As part of this study, we will remove your identifiable, confidential information from the database then share it with foreign collaborators for future analyses.

Your blood sample will be analyzed and stored at the Laboratory for Clinical Research on Acute Febrile Illnesses. Your nasal and throat secretions and feces will be stored at the Laboratory for Respiratory Viruses and Measles. Both labs are located at 4365 Avenida Brasil on the Manguinhos Campus of Fiocruz in Rio de Janeiro. The researchers conducting this study will be responsible for the storage of your samples. You can withdraw consent for the storage of your biological samples whenever you wish. You should let us know in writing that you wish to withdraw, and sign and date your request. Your samples will then be destroyed without affecting your medical care.

Brasil P, *et al. BMJ Open* 2022; 12:e067212. doi: 10.1136/bmjopen-2022-067212

We request your authorization to store your samples for up to ten years. Future studies with the samples will be important for the development of new diagnostics, treatments, and vaccines. Such research will only be carried out following approval by the Brazilian National Research Ethics Committee and/or the Ethics Committee of the National Institute of Infectious Diseases at Fiocruz. It may be necessary for us to seek your free and informed written consent before carrying out such future research. If you do not agree to the use of your biological samples in future studies, your samples will be thrown away at the end of this study.

The greatest risks associated with your participation in this study are those related to: the blood draw, which can cause pain or redness at the site of the puncture; the collection of the throat or nasal swabs, which do not typically cause pain, but may cause light discomfort, cough, nausea, eye watering, the urge to sneeze, or light bleeding. These risks will be minimized because the procedures will be carried out by trained professionals. A nurse will give you one-on-one instructions so that you will not feel uncomfortable when collecting the fecal sample.

Another risk associated with this study is that there could be a loss of confidentiality. However, during the analysis of the data, we will replace your name with a code. Confidential information will not be transmitted over the internet. Your privacy will be guaranteed by the fact that your personal data will be de-identified and the results will be presented in an aggregated format in vehicles of scientific communication.

The benefits that this study may produce are information and guidelines that can improve the detection of SARS-CoV-2 by identifying the age groups that have the greatest risk of

Brasil P, *et al. BMJ Open* 2022; 12:e067212. doi: 10.1136/bmjopen-2022-067212

contracting the virus. This can contribute to the implementation of public health policies and improve scientific knowledge.

Your participation in the study is entirely voluntary and you can refuse to take part or stop at any time without giving us a reason. Your withdrawal will not affect your access to medical care. If you decide not to participate in the study, we will offer you standard medical care including recommendations about public clinics and hospitals where you can receive care later if necessary.

If your SARS-CoV-2 test is positive, you will received all necessary care and medical advice. We guarantee your right to complete medical care free of charge for as long as necessary should any harm befall you due to your participation in this study.

In accordance with Brazilian law, you have the right to seek damages if you are harmed in the study.

At any time during or after the study you have the right to ask the researcher for information regarding your participation in the study. This request can be made via the contact information listed on this form. We guarantee that you will have access to the results of any medical test carried out during the study whenever you or your legal representative requests it. You will also have access to the overall results obtained at the end of the project.

You will not receive financial compensation for participating in this study. All costs associated with the study will be covered by the principal investigator. In other words, you will incur no expense for study procedures or exams.

We have printed two copies of this form, one of which will be made available to you and the other of which will be kept by the researcher. Every page should be initialed by you and either by the researcher in charge or by someone to whom she has delegated responsibility for signing.

For more information, please contact the Principal Investigator of the study: Dr. Patrícia Brasil. Av. Brasil 4365, Instituto Nacional de Infectologia Evandro Chagas – Fiocruz, sala 109, subsolo do hospital. Telephones: 3865-9115 or 3965-9110

Should you have any questions about the ethical conduct of the study, please contact the Committee for Ethics in Research at the National Institute of Infectious Diseases at the Oswaldo Cruz Foundation. The Ethics Committee is a body whose purpose is to defend the interests of research study participants preserving their dignity and personhood while also contributing to the advancement of research that respects ethical norms. Thus, the Committee's role is to evaluate and monitor the conduct of the research project in such a way as to respect ethical principles that respect human rights, individual dignity, autonomy, benevolence, confidentiality, and privacy.

Comitê de Ética em Pesquisa Instituto Nacional de Infectologia Evandro Chagas (INI) Manguinhos - Rio de Janeiro - RJ Tel: 3865-9595 (Business hours: Monday-Friday 9am to 5 pm)

If you wish, you also may contact the Brazilian National Committee for Research Ethics (Conep): Tel: (61) 3315-5878 / (61) 3315-5879. E-Mail: conep@saude.gov.br

I understand all of these instructions and therefore authorize the professionals of Fiocruz to use my blood samples, nasal and throat secretions, and feces while preserving the confidentiality of the results, which will be made available to me individually. The results may also be reported only to public health institutions (municipal and state health departments and the Ministry of Health), and can be published in scientific vehicles of communication.

( ) I also authorize the storage and use of my biological material collected during this study for future studies.

( ) I do not authorize the storage of my samples for future studies.

Date: _____/_____/__________

Name of the participant:

____

Participant's signature:

____

Signature of the researcher responsible:

____

Brasil P, *et al. BMJ Open* 2022; 12:e067212. doi: 10.1136/bmjopen-2022-067212
