# Supplementary material for: Cohort-profile: Household transmission of SARS-CoV-2 in a low-resource community in Rio de Janeiro, Brazil
Source: BMJ Open. 2022 Dec 7;12(12):e067212. doi: 10.1136/bmjopen-2022-067212 (PMC9729844; doi:10.1136/bmjopen-2022-067212)
Supplement: Author's manuscript [file bmjopen-2022-067212.draft_revisions.pdf]

BMJ Open is committed to open peer review. As part of this commitment we make the peer review history of every article we publish publicly available.

When an article is published we post the peer reviewers' comments and the authors' responses online. We also post the versions of the paper that were used during peer review. These are the versions that the peer review comments apply to.

The versions of the paper that follow are the versions that were submitted during the peer review process. They are not the versions of record or the final published versions. They should not be cited or distributed as the published version of this manuscript.

BMJ Open is an open access journal and the full, final, typeset and author-corrected version of record of the manuscript is available on our site with no access controls, subscription charges or pay-per-view fees (http://bmjopen.bmj.com).

If you have any questions on BMJ Open's open peer review process please email info.bmjopen@bmj.com

BMJ Open

Cohort profile: SARS-CoV-2 in a low-resource community in Rio de Janeiro, Brazil

Journal:	*BMJ Open*
Manuscript ID	bmjopen-2022-067212
Article Type:	Cohort profile
Date Submitted by the Author:	08-Aug-2022
Complete List of Authors:	Brasil, Patrícia ; Fundacao Oswaldo Cruz, Instituto Nacional de Infectologia Evandro Chagas<br>Damasceno, Luana; Fundacao Oswaldo Cruz, Instituto Nacional de Infectologia Evandro Chagas<br>Fuller, Trevon; University of California Los Angeles, Institute of the Environment & Sustainability; Fundacao Oswaldo Cruz, Instituto Nacional de Infectologia Evandro Chagas<br>Bastos, Leonardo; Fundacao Oswaldo Cruz, Scientific Computing Program<br>Cruz, Oswaldo; Fundacao Oswaldo Cruz, Scientific Computing Program<br>Medeiros, Fernando; Fundacao Oswaldo Cruz, Instituto Nacional de Infectologia Evandro Chagas<br>CALVET, GUILHERME; Oswaldo Cruz Foundation, Acute Febrile Illnesses Clinical Research Laboratory<br>Resende, Paola; Fundacao Oswaldo Cruz, Laboratory of Respiratory Viruses and Measles<br>Whitworth, Jimmy ; LSHTM,<br>Smith, Chris; London School of Hygiene & Tropical Medicine, Clinical Research Department; Nagasaki University, School of Tropical Medicine and Global Health<br>Siqueira, Marilda; Fundacao Oswaldo Cruz, Laboratório de Vírus Respiratórios e do Sarampo, IOC<br>Carvalho, Marilia; Fundacao Oswaldo Cruz, Scientific Computing Program
Keywords:	COVID-19, Community child health < PAEDIATRICS, Epidemiology < INFECTIOUS DISEASES

SCHOLARONE™
Manuscripts

I, the Submitting Author has the right to grant and does grant on behalf of all authors of the Work (as defined in the below author licence), an exclusive licence and/or a non-exclusive licence for contributions from authors who are: i) UK Crown employees; ii) where BMJ has agreed a CC-BY licence shall apply, and/or iii) in accordance with the terms applicable for US Federal Government officers or employees acting as part of their official duties; on a worldwide, perpetual, irrevocable, royalty-free basis to BMJ Publishing Group Ltd ("BMJ") its licensees and where the relevant Journal is co-owned by BMJ to the co-owners of the Journal, to publish the Work in this journal and any other BMJ products and to exploit all rights, as set out in our licence.

The Submitting Author accepts and understands that any supply made under these terms is made by BMJ to the Submitting Author unless you are acting as an employee on behalf of your employer or a postgraduate student of an affiliated institution which is paying any applicable article publishing charge ("APC") for Open Access articles. Where the Submitting Author wishes to make the Work available on an Open Access basis (and intends to pay the relevant APC), the terms of reuse of such Open Access shall be governed by a Creative Commons licence – details of these licences and which Creative Commons licence will apply to this Work are set out in our licence referred to above.

Other than as permitted in any relevant BMJ Author's Self Archiving Policies, I confirm this Work has not been accepted for publication elsewhere, is not being considered for publication elsewhere and does not duplicate material already published. I confirm all authors consent to publication of this Work and authorise the granting of this licence.

For peer review only

Title: Cohort-profile: SARS-CoV-2 in a low-resource community in Rio de Janeiro, Brazil

Short title: SARS-CoV-2 in a low-resource community in Brazil

Patricia Brasil (ORCID: 0000-0001-9555-7976)[1], Luana Damasceno[1], Trevon Fuller[1,2], Leonardo S. Bastos[3], Oswaldo G. Cruz[3], Fernando Medeiros[1], Guilherme Calvet[1], Paola Cristina Resende[4], Jimmy Whitworth[5], Chris Smith[5], Marilda Siqueira[4], Marilia Carvalho[3]

[1]Acute Febrile Illnesses Department, Evandro Chagas National Institute of Infectious Diseases, Oswaldo Cruz Foundation, Rio de Janeiro, Brazil

[2]Institute of the Environment and Sustainability, University of California Los Angeles, Los Angeles, USA

[3]Scientific Computing Program, Oswaldo Cruz Foundation, Rio de Janeiro, Brazil

[4]Laboratory of Respiratory Viruses and Measles, Oswaldo Cruz Foundation, Rio de Janeiro, Brazil

[5]Department of Clinical Research, London School of Hygiene and Tropical Medicine, London, UK

Correspondence to: Dr. Patrícia Brasil, MD, PhD. Oswaldo Cruz Foundation, Avenida Brasil, 4365 Manguinhos, Rio de Janeiro - RJ, 21040-900 Brazil; E-mail: patricia.brasil@ini.fiocruz.br

Word count: 2,533

For peer review only

ABSTRACT

Purpose: To better understand the local transmission of SARS-COV-2 in low-resource communities and the role of children in such transmission, we have followed a cohort of children and their household contacts in Rio de Janeiro during the COVID-19 pandemic (2020 to 2022).

Participants: This is an open prospective cohort study of children ≤ 12 years old and their household contacts. We enrolled 842 families from May 20, 2020 to May 31, 2022. This period encompassed different locally-circulating SARS-CoV-2 lineages, in particular, B.1.1.33 during the first wave followed by variants Zeta (P.2), Gamma (P.1/P.1.*), Delta (B.1.617.2/AY.*), and Omicron (BA.*). The average duration of follow-up per family was approximately 250 days. The average number of residents per household was four. Among adults, the majority of participants were women (62%).

Findings to date: The seroprevalence of SARS-CoV-2 was approximately 20% from May 2020 to June 2021 but increased to more than 80% for most age groups with the rollout of vaccination. During the first wave, children were not the principal source of SARS-CoV-2 infections in their households and household crowding was significantly associated with SARS-CoV-2 incidence. Through the Delta wave, the incidence among children was lower than that of older participants. Subsequently, vaccination of the elderly reached 90% and incidence among children was higher.

Future plans: Future studies will investigate cellular and humoral immune responses to locally-circulating SARS-CoV-2 variants, which is relevant for the design of vaccines, antivirals, and monoclonal antibodies. We will also engage in outreach to encourage vaccination as a means of limiting the transmission of novel SARS-CoV-2 variants and other emerging pathogens.

Keywords: COVID-19; community child health; epidemiology

For peer review only - http://bmjopen.bmj.com/site/about/guidelines.xhtml

INTRODUCTION

The emergence of Coronavirus disease 2019 (COVID-19) has caused a serious health crisis affecting the world since 2020. More than 520 million confirmed cases, including 6.3 million deaths, have been reported to date worldwide.[1] In the Western Hemisphere, more than 20% of all COVID-19 cases have occurred in Brazil. At the global scale, 10% of COVID-19 deaths have been in Brazil. Rio de Janeiro has reported the second highest number of COVID-19 deaths of any city in Brazil.[2,3]

The number of COVID-19 cases and deaths are believed to have been grossly underestimated, particularly in the poorest regions. Several studies have shown an association between the poorest cities or neighbourhoods and the COVID-19 burden. Inequities in access to health services are hypothesized to be the main driver of deaths.[4,5] However, much of the research investigating this question has been based upon cross-sectional studies.[6] This has limited our ability to draw robust conclusions about the causes of COVID-19 morbimortality.

Another gap in our knowledge about COVID-19 is that there is scant data about household transmission in low- and middle-income countries. Among the factors that may drive such transmission are the difficulty of remaining isolated in crowded households in low-resource communities and insufficient access to laboratory tests. In addition, transmission patterns have evolved in the two years of the pandemic with circulation of SARS-CoV-2 variants, which differ in virulence and transmissibility. On the other hand, the availability of tests, especially the rapid antigen test, has improved the detection of new infections.

For peer review only - http://bmjopen.bmj.com/site/about/guidelines.xhtml

To better understand the local transmission of SARS-COV-2 in the aforementioned communities and the role of children in such transmission, we have followed a cohort of children and their household contacts during the COVID-19 pandemic period (2020-2022). We have tested the cohort participants at regular intervals for SARS-CoV-2 via RT-PCR of nasopharyngeal swabs and anti-spike IgG antibody tests of serum samples.

COHORT DESCRIPTION

This is an open prospective cohort study of children ≤ 12 years old and their household contacts. The present report describes our findings for the period from May 20, 2020 to May 31, 2022. This period encompassed different locally-circulating SARS-CoV-2 lineages, in particular, B.1.1.33 during the first wave[7] followed by variants Zeta (P.2), Gamma (P.1/P.1.*), Delta (B.1.617.2/AY.*), and Omicron (BA.*). The epidemiologic scenario in the community has evolved during the study. In March 2020, social distancing policies including quarantine and school closure were implemented. From April 2020 to October 2021, emergency financial assistance was made available to low-income households. In June 2020, bars, stores, and restaurants were reopened. However, adherence to control measures varied among neighbourhoods and was generally poorest in low-resource communities. Vaccination began in Rio de Janeiro in January 2021. Public schools resumed in-person classes in November 2021, immediately before the end of the academic year.

Recruitment took place at the Germano Sinval Faria Health Center, at the Sergio Arouca National School of Public Health, Oswaldo Cruz Foundation. This primary health care centre, located in

For peer review only - http://bmjopen.bmj.com/site/about/guidelines.xhtml

Manguinhos, a neighbourhood in the Northern sector of the city of Rio de Janeiro, provides primary care services to residents of the Manguinhos complex, all of which have very low household income. The clinic provides medical care free of charge to people who live in the complex serving an average of 30,000 adults and 4,300 children ≤12 years old every month. Children who were brought to the primary local health centre for immunization, check-ups or emergency care were invited to participate in the study, irrespective of whether or not they were symptomatic. After written informed consent was provided by a parent or legal guardian, the child was enrolled and nasopharyngeal and serum samples were collected. We also obtained the street address of the child's home, the parent or guardian's workplace (if different), and the telephone numbers of any relatives. This information was used to schedule home visits.

For peer

In each household, we collect samples on Days 1 (first visit), 14 and 28, every three months during the first year and twice in the second year. All symptomatic participants were evaluated and provided with medical care until symptoms resolved. The total follow-up time for each family was 24 months. At households with any symptomatic resident, an additional sample was collected on Day 7. Households in which no individual was examined during the entirety of a given epidemic wave were considered lost to follow-up after eight unsuccessful contact attempts by telephone and smartphone messaging apps. Individuals in each household who missed one or more study visits were excluded only if the study team was unable to contact the participant during the entire wave.

For peer review only - http://bmjopen.bmj.com/site/about/guidelines.xhtml

The screening of participants, the informed consent process, and data capture of the enrolled participants was carried out with REDCap.[8,9] The information collected at baseline and each subsequent visit are summarized below:

- Sociodemographic characteristics: age, education, ethnicity, profession; residence: address, GPS coordinates, telephone, number of people living in the household, and number of rooms.
- Behavioural data: mask use, use of public transportation, visits to restaurants, bars, malls, movie theatres, cookouts, birthday parties, or other events where there was a crowd.
- Clinical manifestations in children: seizures, anosmia, dysgeusia, food refusal, fever, cough, nasal congestion, prostration, shortness of breath, abdominal pain, and diarrhoea.
- Adolescent/adult clinical variables: anosmia, dysgeusia, loss of appetite, fever, cough, rhinorrhoea, headache, prostration, shortness of breath, sore throat, abdominal pain, and diarrhoea.
- Vaccination status: number of doses, manufacturer, and vaccination date.
- Laboratory test results: SARS-CoV-2 RT-PCR test, SARS-CoV-2 lineage genomic test, and SARS-CoV-2 IgG antibody test of serum samples.

The structure of the participant database is shown in Figure 1.

During the enrolment visit, a study nurse administered a questionnaire to the child's parent or guardian to collect data about the child's socio-demographic and clinical characteristics and medical history, as well as characteristics of the household. The questionnaire was completed on a tablet and took approximately 20 minutes. If the child was symptomatic an additional clinical questionnaire was completed by a paediatrician requiring approximately 30 more minutes.

For peer review only
For peer review only - http://bmjopen.bmj.com/site/about/guidelines.xhtml

Home visits were scheduled by phone and conducted by a team of field workers, including paediatricians and nurses. During these visits, study participation was offered to the child's household contacts, nasopharyngeal and serum samples were collected, and the GPS coordinates of the household were recorded. The study personnel completed a questionnaire for each contact who consented to participate consisting of clinical history, sociodemographic characteristics, and vaccination status. Visits lasted an average of 40 minutes. If time constraints prevented a participant from responding to all of the questions during the visit, the study team followed up with him or her by phone to complete the questionnaire.

Nasopharyngeal swabs were tested by real time RT-PCR to amplify the E gene and the RdRp region of the Orf1ab gene of SARS-CoV-2 (Charité/Berlin, Germany). Cycle thresholds (CT) less than 40 were classified as positive. We assembled SARS-CoV-2 genomes from Illumina amplicons using the CLC Genomics Workbench v 20.0.4 (QIAGEN). Phylogenetic relationships among SARS-CoV-2 samples were determined using the Pango Lineages tool. SARS-CoV-2 serology (IgG) was performed using a Microparticle Chemiluminescent Immunoassay (CMIA), targeting the S gene (Abbott Laboratories, Abbott Park, IL, USA). All assays were performed according to the manufacturers' instructions.

For peer review only

FINDINGS TO DATE

In total, 79.5% of the individuals whom we approached consented to participate in the study. Those who were approached but did not enrol in the study were either adults who stated that they did not have time to participate or children whose parent or legal guardian was not present and therefore

For peer review only - http://bmjopen.bmj.com/site/about/guidelines.xhtml

the consent could not be obtained. A total of 842 families were enrolled in the study: 34.4% in the first epidemic wave, 16.4% in the Zeta wave, and 23.9% in the Gamma wave, 18.3% in the Delta wave, and 7% in the Omicron wave. A total of 7,091 RTPCR and 5,554 serology tests were performed.

The average number of participants recruited per week was 27.1. The period of follow-up was 18 months. As enrolment began in May 2022 and we followed participants through May 2022, some participants had completed their schedule of visits before the end of the study (Figure 2). Those who were recruited recently have not completed all of their follow-up visits. Currently, 67.5% of the participants who were enrolled are still being followed. Among 7,459 home visits, 15.3% constituted one-off visits. In these cases, subsequent home visits outside the scheduled visit window may still occur. The mean follow-up time was 248 days for participants ≥ 13 years old and 265 days for those ≤ 12 years old. The mean follow-up time was greater for women ≥ 13 years old (152 days) than for men in the same age group (112 days).

The sociodemographic characteristics of the families recruited were similar across the epidemic waves. The average number of residents per household was four. The majority of participants were women (62%), reflecting not only the recruitment strategy, as children are usually accompanied by their mothers or grandmothers, but also the caregiver at home. Across all recruitment periods, 45-54% of the participants were adults and 36-52% were children ≤ 12 years old (Table 1).

For peer review only - http://bmjopen.bmj.com/site/about/guidelines.xhtml

Table 1. Demographic characteristics of the cohort participants by epidemic wave.

Characteristic		Overall N=1,773	B.1.1.33 N=624	Zeta N=343	Gamma N=463	Delta N=291	Omicron N=52
Median days of follow-up (IQR)		224 (109-368)	320 (196-526)	266 (150-426)	168 (70-259)	137 (49-203)	78 (21-123)
Median age in years (IQR)		16 (7-35)	14 (5-36)	19 (7-35)	19 (8-38)	15 (8-32)	9 (4-32)
		**N (%)**	**N (%)**	**N (%)**	**N (%)**	**N (%)**	**N (%)**
Male sex (%)		671 (38%)	238 (38%)	131 (38%)	173 (37%)	110 (38%)	19 (37%)
Age (years)	0-12	644 (38%)	241 (41%)	119 (36%)	150 (34%)	109 (38%)	25 (52%)
	13-18	209 (12%)	63 (11%)	33 (10%)	65 (15%)	47 (17%)	1 (2%)
	≥19	841 (50%)	284 (48%)	175 (54%)	232 (52%)	128 (45%)	22 (46%)

The median age of the participants was 14-19 during the first four waves of the participants but decreased to nine years of age during the Omicron wave. Across all five waves of the pandemic, the interquartile range of participant ages was approximately five years to thirty-five years old. The seroprevalence of SARS-CoV-2 antibodies indicative of past exposure to the virus or a

For peer review only - http://bmjopen.bmj.com/site/about/guidelines.xhtml

vaccine was approximately 20% from May 2020 to June 2021. Seroprevalence subsequently increased precipitously, likely reflecting the rollout of SARS-CoV-2 vaccination (Figure 3).

Complete vaccination (two doses of BNT162b2, AZD1222, CoronaVac (Sinovac), or one dose of Ad26.COV2.S) increased with age and was over 80% for all but the youngest age groups (Figure 4).

The first published report from this cohort provided evidence that children were not the principal source of SARS-CoV-2 infections in their households during the first wave of the pandemic, but more frequently acquired the virus from adolescent and adult household contacts.[10] In a subsequent study, we estimated the incidence of SARS-CoV-2 in the cohort during the B.1.1.33, Zeta, Gamma, and Delta waves[11]. Household crowding was significantly associated with SARS-CoV-2 incidence only during the first wave[11]. Through the Delta wave, the incidence among children was lower than that of older participants. Subsequently, vaccination of the elderly reached 90% and incidence among children was higher.

PATIENT AND PUBLIC INVOLVEMENT

The study personnel include residents of Manguinhos who were first hired during our previous cohort studies.[10 12-15] These professionals continuously provide input about the type of data collection that is feasible in this community afflicted by armed conflict, food insecurity, and high unemployment. All study participants were given the opportunity to provide feedback about the design of the study during the first home visit. We report COVID-19 prevalence rates in the community in real-time via a publicly-available dashboard.

For peer review only - http://bmjopen.bmj.com/site/about/guidelines.xhtml

STRENGTHS AND LIMITATIONS OF THIS STUDY

The study's high rates of participant retention and visit completion are among its principal strengths. Furthermore, since we recruited asymptomatic and symptomatic participants, we were able to estimate the overall incidence in this population, not restricted to individuals with obvious clinical signs of COVID-19. Our study data are unique because this is a community that is not easily accessible to researchers due to high levels of poverty and violence. Nevertheless, it is emblematic of low-resource communities in other cities in Latin America. Our study team included personnel from different disciplines including epidemiology, medicine, virology, immunology, and statistics. We believe that this strengthened the research because we were able to examine the ramifications of the COVID-19 pandemic for this community from a number of different angles. Finally, since the cohort has been followed continuously for two years, we have been able to gather data about successive variants that have swept through the population.

A limitation of our study is that it was sometimes necessary to reschedule home visits due to armed conflicts in the community. As such, we could have missed acute SARS-CoV-2 infections that occurred during cancelled study visits. Another important caveat is that due to logistic constraints it was not possible for us to externally validate the study findings in other communities. Finally, loss to follow-up increased and recruitment decreased with the relaxation of social distancing policies because eligible adults resumed working outside the community and were not able to participate in the study.

For peer review only - http://bmjopen.bmj.com/site/about/guidelines.xhtml

COLLABORATION

While the data at the individual level are not openly available to preserve the anonymity of the study participants, researchers who are interested in potential collaboration should contact the corresponding author. The study steering committee will evaluate data requests in accordance with best practices in cohort studies to ensure that data sharing would preserve confidentiality and be compatible with the terms of the Informed Consent Form used in this study.

FUTURE PLANS

The biobank of cryopreserved peripheral blood mononuclear cells (PBMC) and serum from the cohort participants will allow future studies of cellular and humoral immune responses to locally-circulating SARS-CoV-2 variants, which is relevant for the design of future vaccines, antivirals, and monoclonal antibodies. We will also analyse the immune profile of participants who experienced breakthrough infections following vaccination. Of course, in order for novel SARS-CoV-2 variants and other emerging pathogens to be controlled, it will be necessary to reduce transmission. To this end, future work will also involve publicizing virus control and prevention practices. We thus plan to create a short 'edutainment' style video to disseminate our results and encourage SARS-CoV-2 immunization. The target audience will be children and families. As part of these efforts, we will work closely with media partners, policymakers and the cohort participants to develop effective outreach strategies. We believe such activities might help bring about better compliance with COVID-19 control measures not only in this community, but also at the regional and global levels.

COMPETING INTERESTS

For peer review only - http://bmjopen.bmj.com/site/about/guidelines.xhtml

All authors declare no competing interests.

ACKNOWLEDGMENTS

Thanks are due to the families in Manguinhos who kindly agreed to participate in the study. This research would not have been possible without the support of Carlos Alberto de Moraes Costa, Iris Maria da Silva Lordello, and the staff of the Germano Sinval Faria Health Center, the Oswaldo Cruz Foundation's COVID-19 Diagnosis Support Unit, Vice-Presidency of Health Production and Innovation, and LabiExames.

FUNDING

Ministério da Ciência, Tecnologia e Inovação Conselho Nacional de Desenvolvimento Científico e Tecnológico 307282/2017-1, Fundação Carlos Chagas Filho de Amparo à Pesquisa do Estado do Rio de Janeiro E-26/202.862/2018, E-26/211.565/2019, E-26/210.149/2020; UK Research and Innovation MR/V033530/1.

ETHICS APPROVAL

The study was approved by the Brazilian National Ethics Committee (CONEP) under protocol number 30639420.0.0000.5262.All participants gave their informed consent to participate in the study.

CONTRIBUTORS

Study concept: P. B., M. C. Collected primary data and contributed to data analysis: L. D. Performed analysis and interpretation of data: O. G. C., L. S. B., M. C., G. C., C. S., J. W.

For peer review only

Conducted the laboratory investigation: P. C. R., M. S. All authors provided intellectual contributions to the manuscript and approved the final manuscript.

REFERENCES

1. World Health Organization. News Release: 14.9 million excess deaths associated with the COVID-19 pandemic in 2020 and 2021. Geneva: Switzerland. Geneva: WHO 2022.
2. Brazilian Ministry of Health. Brazilian Coronavirus Dashboard. Available at: https://covid.saude.gov.br/ Accessed July 26, 2022. Brasília: Brazilian Ministry of Health 2022.
3. World Health Organization. Coronavirus (COVID-19) Dashboard. Available at: https://covid19.who.int/. Accessed 26 July 2022. Geneva: WHO 2022.
4. Silva LL, Carvalho Dutra Ad, Andrade Ld, et al. Emergency care gap in Brazil: geographical accessibility as a proxy of response capacity to tackle COVID-19. *Frontiers in Public Health* 2021;9 doi: 10.3389/fpubh.2021.740284
5. Li SL, Pereira RHM, Prete Jr CA, et al. Higher risk of death from COVID-19 in low-income and non-White populations of São Paulo, Brazil. *BMJ Global Health* 2021;6(4):e004959. doi: 10.1136/bmjgh-2021-004959
6. Mackey K, Ayers CK, Kondo KK, et al. Racial and ethnic disparities in COVID-19-related infections, hospitalizations, and deaths a systematic review. *Annals of Internal Medicine* 2021;174(3):362. doi: 10.7326/m20-6306
7. Resende PC, Delatorre E, Gräf T, et al. Evolutionary dynamics and dissemination pattern of the SARS-CoV-2 lineage B.1.1.33 during the early pandemic phase in Brazil. *Frontiers in Microbiology* 2021;11 doi: 10.3389/fmicb.2020.615280

For peer review only - http://bmjopen.bmj.com/site/about/guidelines.xhtml

8. Harris PA, Taylor R, Thielke R, et al. Research electronic data capture (REDCap)--a metadata-driven methodology and workflow process for providing translational research informatics support. *Journal of Biomedical Informatics* 2009;42(2):377-81. doi: 10.1016/j.jbi.2008.08.010

9. Harris PA, Taylor R, Minor BL, et al. The REDCap consortium: building an international community of software platform partners. *Journal of Biomedical Informatics* 2019;95:103208.

10. Lugon P, Fuller T, Damasceno L, et al. SARS-CoV-2 infection dynamics in children and household contacts in a slum in Rio de Janeiro. *Pediatrics* 2021;148(1) doi: 10.1542/peds.2021-050182

11. Carvalho MS, Bastos LS, Fuller T, et al. Incidence of SARS-CoV-2 over four epidemic waves in a low-resource community in Rio de Janeiro, Brazil: A prospective cohort study. *The Lancet Regional Health – Americas* 2022;12 doi: 10.1016/j.lana.2022.100283

12. Pedro RS, Carvalho MS, Girianelli VR, et al. A populational-based birth cohort study in a low-income urban area in Rio de Janeiro, Brazil: implementation and description of the characteristics of the study. *Cadernos de Saúde Pública* 2019;35

13. de Aguiar DF, de Barros ENC, Ribeiro GS, et al. A prospective, multicentre, cohort study to assess the incidence of dengue illness in households from selected communities in Brazil (2014-2018). *International Journal of Infectious Diseases*2021;108:443-53. doi: 10.1016/j.ijid.2021.04.062 [published Online First: 2021/04/25]

14. Damasceno L, Terzian ACB, Fuller T, et al. Why did ZIKV perinatal outcomes differ in distinct regions of Brazil? An exploratory study of two cohorts. *Viruses* 2021;13(5):736.

For peer review only - http://bmjopen.bmj.com/site/about/guidelines.xhtml

15. Brasil P, Pereira JP, Moreira ME, et al. Zika virus infection in pregnant women in Rio de Janeiro. *New England Journal of Medicine* 2016;375(24):2321-34. doi: 10.1056/NEJMoa1602412

Figure Captions

Figure 1. Cohort database structure.

Figure 2. Recruitment and retention by SARS-CoV-2 epidemic wave.

Figure 3. SARS-CoV-2 seroprevalence by month of sample collection.

Figure 4. SARS-CoV-2 vaccine coverage by age group.

For peer review only

For peer review only - http://bmjopen.bmj.com/site/about/guidelines.xhtml

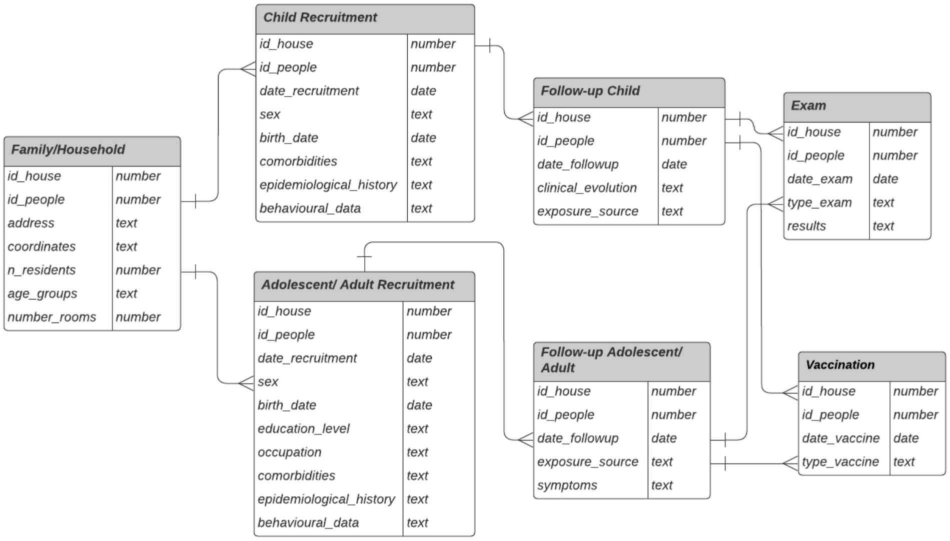

Cohort database structure.

548x324mm (63 x 63 DPI)

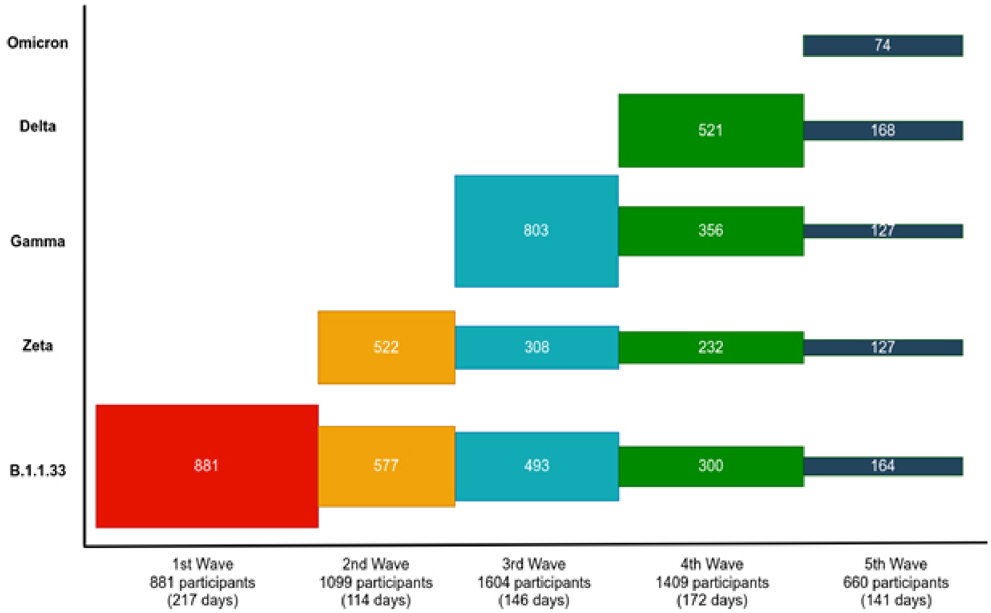

Recruitment and retention by SARS-CoV-2 epidemic wave.

220x140mm (72 x 72 DPI)

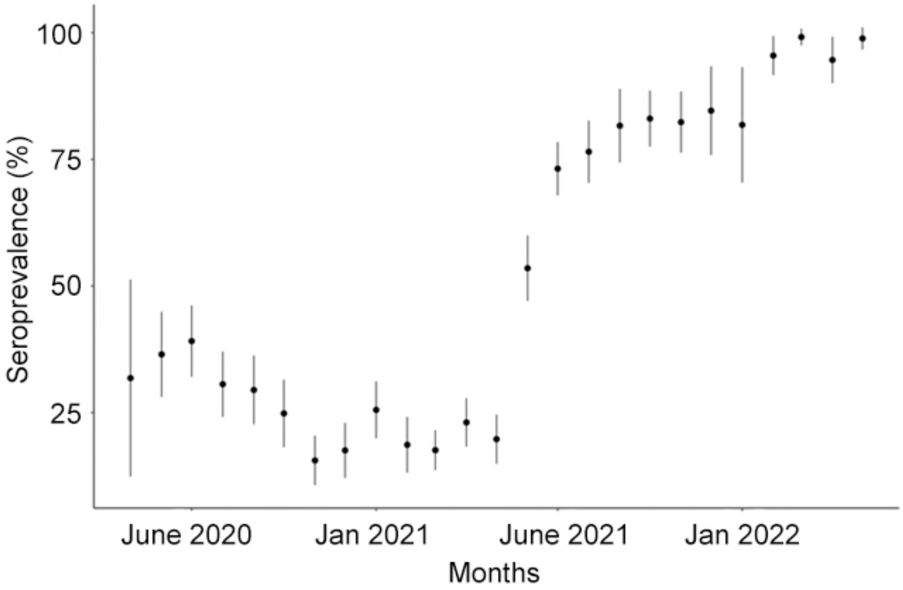

SARS-CoV-2 seroprevalence by month of sample collection.

643x392mm (28 x 28 DPI)

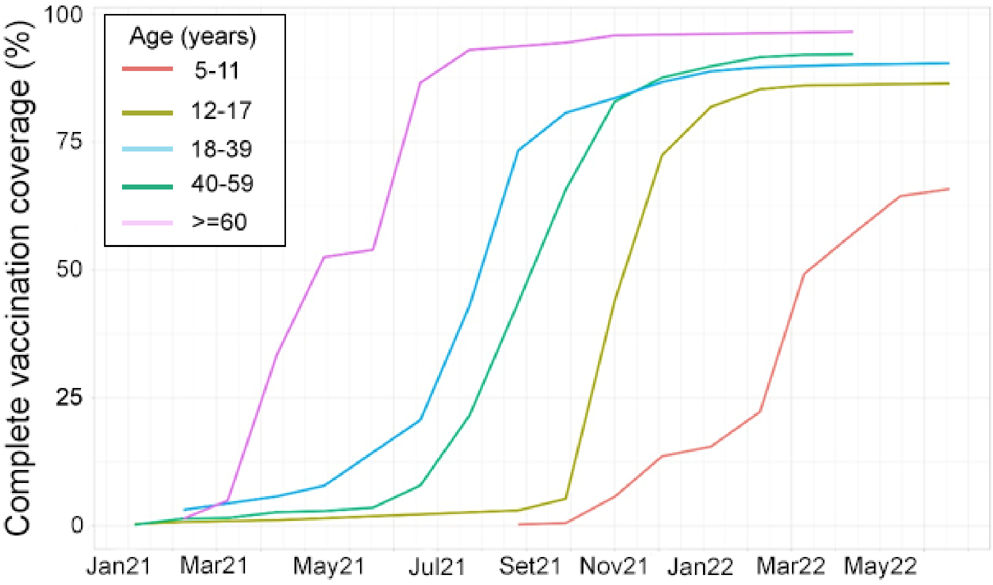

SARS-CoV-2 vaccine coverage by age group.

552x392mm (28 x 28 DPI)

STROBE 2007 (v4) Statement—Checklist of items that should be included in reports of *cohort studies*

Section/Topic	Item #	Recommendation	Reported on page #
**Title and abstract**	1	(*a*) Indicate the study's design with a commonly used term in the title or the abstract	1
		(*b*) Provide in the abstract an informative and balanced summary of what was done and what was found	2
**Introduction**			
Background/rationale	2	Explain the scientific background and rationale for the investigation being reported	3
Objectives	3	State specific objectives, including any prespecified hypotheses	4
**Methods**			
Study design	4	Present key elements of study design early in the paper	4
Setting	5	Describe the setting, locations, and relevant dates, including periods of recruitment, exposure, follow-up, and data collection	4
Participants	6	(*a*) Give the eligibility criteria, and the sources and methods of selection of participants. Describe methods of follow-up	5
		(*b*) For matched studies, give matching criteria and number of exposed and unexposed	NA
Variables	7	Clearly define all outcomes, exposures, predictors, potential confounders, and effect modifiers. Give diagnostic criteria, if applicable	7
Data sources/ measurement	8*	For each variable of interest, give sources of data and details of methods of assessment (measurement). Describe comparability of assessment methods if there is more than one group	6
Bias	9	Describe any efforts to address potential sources of bias	5
Study size	10	Explain how the study size was arrived at	8
Quantitative variables	11	Explain how quantitative variables were handled in the analyses. If applicable, describe which groupings were chosen and why	6
Statistical methods	12	(*a*) Describe all statistical methods, including those used to control for confounding	NA
		(*b*) Describe any methods used to examine subgroups and interactions	NA
		(*c*) Explain how missing data were addressed	NA
		(*d*) If applicable, explain how loss to follow-up was addressed	5
		(*e*) Describe any sensitivity analyses	NA
**Results**			

For peer review only

Participants	13*	(a) Report numbers of individuals at each stage of study—eg numbers potentially eligible, examined for eligibility, confirmed eligible, included in the study, completing follow-up, and analysed	7
		(b) Give reasons for non-participation at each stage	8
		(c) Consider use of a flow diagram	Figure 1
Descriptive data	14*	(a) Give characteristics of study participants (eg demographic, clinical, social) and information on exposures and potential confounders	9
		(b) Indicate number of participants with missing data for each variable of interest	NA
		(c) Summarise follow-up time (eg, average and total amount)	8
Outcome data	15*	Report numbers of outcome events or summary measures over time	9
Main results	16	(*a*) Give unadjusted estimates and, if applicable, confounder-adjusted estimates and their precision (eg, 95% confidence interval). Make clear which confounders were adjusted for and why they were included	NA
		(*b*) Report category boundaries when continuous variables were categorized	5
		(*c*) If relevant, consider translating estimates of relative risk into absolute risk for a meaningful time period	NA
Other analyses	17	Report other analyses done—eg analyses of subgroups and interactions, and sensitivity analyses	NA
**Discussion**			
Key results	18	Summarise key results with reference to study objectives	10
**Limitations**			
Interpretation	20	Give a cautious overall interpretation of results considering objectives, limitations, multiplicity of analyses, results from similar studies, and other relevant evidence	11
Generalisability	21	Discuss the generalisability (external validity) of the study results	10
**Other information**			
Funding	22	Give the source of funding and the role of the funders for the present study and, if applicable, for the original study on which the present article is based	13

*Give information separately for cases and controls in case-control studies and, if applicable, for exposed and unexposed groups in cohort and cross-sectional studies.

Note: An Explanation and Elaboration article discusses each checklist item and gives methodological background and published examples of transparent reporting. The STROBE checklist is best used in conjunction with this article (freely available on the Web sites of PLoS Medicine at http://www.plosmedicine.org/, Annals of Internal Medicine at http://www.annals.org/, and Epidemiology at http://www.epidem.com/). Information on the STROBE Initiative is available at www.strobe-statement.org.

For peer review only

BMJ Open

Cohort-profile: Household transmission of SARS-CoV-2 in a low-resource community in Rio de Janeiro, Brazil

Journal:	*BMJ Open*
Manuscript ID	bmjopen-2022-067212.R1
Article Type:	Cohort profile
Date Submitted by the Author:	24-Oct-2022
Complete List of Authors:	Brasil, Patrícia ; Fundacao Oswaldo Cruz, Instituto Nacional de Infectologia Evandro Chagas<br>Damasceno, Luana; Fundacao Oswaldo Cruz, Instituto Nacional de Infectologia Evandro Chagas<br>Fuller, Trevon; University of California Los Angeles, Institute of the Environment & Sustainability; Fundacao Oswaldo Cruz, Instituto Nacional de Infectologia Evandro Chagas<br>Bastos, Leonardo; Fundacao Oswaldo Cruz, Scientific Computing Program<br>Cruz, Oswaldo; Fundacao Oswaldo Cruz, Scientific Computing Program<br>Medeiros, Fernando; Fundacao Oswaldo Cruz, Instituto Nacional de Infectologia Evandro Chagas<br>CALVET, GUILHERME; Oswaldo Cruz Foundation, Acute Febrile Illnesses Clinical Research Laboratory<br>Resende, Paola; Fundacao Oswaldo Cruz, Laboratory of Respiratory Viruses and Measles<br>Whitworth, Jimmy ; LSHTM,<br>Smith, Chris; London School of Hygiene & Tropical Medicine, Clinical Research Department; Nagasaki University, School of Tropical Medicine and Global Health<br>Siqueira, Marilda; Fundacao Oswaldo Cruz, Laboratório de Vírus Respiratórios e do Sarampo, IOC<br>Carvalho, Marilia; Fundacao Oswaldo Cruz, Scientific Computing Program
**Primary Subject Heading**:	Infectious diseases
Secondary Subject Heading:	Epidemiology
Keywords:	COVID-19, Community child health < PAEDIATRICS, Epidemiology < INFECTIOUS DISEASES

SCHOLARONE™
Manuscripts

I, the Submitting Author has the right to grant and does grant on behalf of all authors of the Work (as defined in the below author licence), an exclusive licence and/or a non-exclusive licence for contributions from authors who are: i) UK Crown employees; ii) where BMJ has agreed a CC-BY licence shall apply, and/or iii) in accordance with the terms applicable for US Federal Government officers or employees acting as part of their official duties; on a worldwide, perpetual, irrevocable, royalty-free basis to BMJ Publishing Group Ltd ("BMJ") its licensees and where the relevant Journal is co-owned by BMJ to the co-owners of the Journal, to publish the Work in this journal and any other BMJ products and to exploit all rights, as set out in our licence.

The Submitting Author accepts and understands that any supply made under these terms is made by BMJ to the Submitting Author unless you are acting as an employee on behalf of your employer or a postgraduate student of an affiliated institution which is paying any applicable article publishing charge ("APC") for Open Access articles. Where the Submitting Author wishes to make the Work available on an Open Access basis (and intends to pay the relevant APC), the terms of reuse of such Open Access shall be governed by a Creative Commons licence – details of these licences and which Creative Commons licence will apply to this Work are set out in our licence referred to above.

Other than as permitted in any relevant BMJ Author's Self Archiving Policies, I confirm this Work has not been accepted for publication elsewhere, is not being considered for publication elsewhere and does not duplicate material already published. I confirm all authors consent to publication of this Work and authorise the granting of this licence.

For peer review only

Title: Cohort-profile: Household transmission of SARS-CoV-2 in a low-resource community in Rio de Janeiro, Brazil

Short title: SARS-CoV-2 in a low-resource community in Brazil

Patricia Brasil (ORCID: 0000-0001-9555-7976)[1], Luana Damasceno[1], Trevon Fuller[1,2], Leonardo S. Bastos[3], Oswaldo G. Cruz[3], Fernando Medeiros[1], Guilherme Calvet[1], Paola Cristina Resende[4], Jimmy Whitworth[5], Chris Smith[5], Marilda Siqueira[4], Marilia Carvalho[3]

[1]Acute Febrile Illnesses Department, Evandro Chagas National Institute of Infectious Diseases, Oswaldo Cruz Foundation, Rio de Janeiro, Brazil

[2]Institute of the Environment and Sustainability, University of California Los Angeles, Los Angeles, USA

[3]Scientific Computing Program, Oswaldo Cruz Foundation, Rio de Janeiro, Brazil

[4]Laboratory of Respiratory Viruses and Measles, Oswaldo Cruz Foundation, Rio de Janeiro, Brazil

[5]Department of Clinical Research, London School of Hygiene and Tropical Medicine, London, UK

Correspondence to: Dr. Patrícia Brasil, MD, PhD. Oswaldo Cruz Foundation, Avenida Brasil, 4365 Manguinhos, Rio de Janeiro - RJ, 21040-900 Brazil; E-mail: patricia.brasil@ini.fiocruz.br

Word count: 2,602

For peer review only - http://bmjopen.bmj.com/site/about/guidelines.xhtml

ABSTRACT

Purpose: To better understand the household transmission of SARS-COV-2 in a low-resource community in Rio de Janeiro during the COVID-19 pandemic (2020 to 2022).

Participants: This is an open prospective cohort study of children ≤ 12 years old and their household contacts. During home visits over 24 months we collected data on sociodemographic characteristics, behavioural data, clinical manifestations of SARS-CoV-2, vaccination status, SARS-CoV-2 RT-PCR and anti-S antibody tests. Among adults, the majority of participants were women (62%).

Findings to date: We enrolled 845 families from May 2020 to May 2022. The median number of residents per household was four. The median household density, defined as the number of persons per room, was 0.95. The risk of SARS-CoV-2 occurrence was higher in households with a high number of persons per room. Children were not the principal source of SARS-CoV-2 infections in their households during the first wave of the pandemic.

Future plans: Future studies will investigate cellular and humoral immune responses to locally circulating SARS-CoV-2 variants, which is relevant for the design of vaccines, antivirals, and monoclonal antibodies. We will also engage in outreach to encourage vaccination as a means of limiting the transmission of novel SARS-CoV-2 variants and other emerging pathogens.

Strengths and Limitations:

- Rates of participant retention and visit completion were high when compared with similar settings.
- We were able to estimate the overall incidence of SARS-Co-V2 infection in this population, not restricted to individuals with obvious clinical signs of COVID-19.
- Since the cohort has been followed continuously for two years, we have been able to gather data about successive variants that have swept through the population.

For peer review only - http://bmjopen.bmj.com/site/about/guidelines.xhtml

- There was some irregularity of study visits, which sometimes had to be rescheduled due to armed conflicts in the community.
- We could have missed acute SARS-CoV-2 infections either between visits or when visits were cancelled.

Keywords: COVID-19, Community child health < PAEDIATRICS, Epidemiology < INFECTIOUS DISEASES

For peer review only

INTRODUCTION

The emergence of Coronavirus disease 2019 (COVID-19) has caused a serious health crisis affecting the world since 2020. More than 624 million confirmed cases, including 6.6 million deaths, have been reported up to October 2022 worldwide.[1] In the Western Hemisphere, more than 20% of all COVID-19 cases have occurred in Brazil. At the global scale, 10% of COVID-19 deaths have been in Brazil. Rio de Janeiro has reported the second highest number of COVID-19 deaths of any city in Brazil.[2 3]

Understanding intrafamily transmission is essential for designing appropriate interventions. Much of the research investigating this question has been based upon cross-sectional studies that do not accompany participants longitudinally making it difficult to investigate the transmission process over time.[4] Longitudinal data about household transmission are particularly scant in low- and middle-income countries. Among the factors that may drive such transmission are the difficulty of remaining isolated in crowded households and insufficient access to laboratory tests. In addition, transmission patterns have evolved in the two years of the pandemic with circulation of SARS-CoV-2 variants, which differ in virulence and transmissibility.

For peer review only - http://bmjopen.bmj.com/site/about/guidelines.xhtml

To better understand the local transmission of SARS-COV-2 in the aforementioned communities and the role of children in such transmission, we have followed a cohort of children and their household contacts during the COVID-19 pandemic period (2020-2022).

COHORT DESCRIPTION

This is an open prospective cohort study of children ≤ 12 years old and their household contacts. The present report describes our findings from May 20, 2020, to May 31, 2022. Recruitment was closed at the end of this analysis, but it is immediately reopened whenever a new variant of concern is detected in the community.

The epidemic context

This period encompassed different locally-circulating SARS-CoV-2 lineages, in particular, B.1.1.33 during the first wave[5] followed by variants Zeta (P.2), Gamma (P.1/P.1.*), Delta (B.1.617.2/AY.*), and Omicron (BA.*). The epidemiologic scenario in the community has evolved during the study.[6] In March 2020, social distancing policies including quarantine and school closure were implemented. From April 2020 to October 2021, emergency financial assistance was made available to low-income households. In June 2020, bars, stores, and restaurants were reopened. However, adherence to control measures varied among neighbourhoods and was generally poorest in low-resource communities. Vaccination began in Rio de Janeiro in January 2021. Public schools resumed in-person classes in November 2021, immediately before the end of the academic year.

Recruitment and eligibility criteria

Recruitment took place at the Germano Sinval Faria Health Centre, at the Sergio Arouca National School of Public Health, Oswaldo Cruz Foundation. This primary health care centre is located in

For peer review only - http://bmjopen.bmj.com/site/about/guidelines.xhtml

Manguinhos, a neighbourhood in the Northern sector of the city of Rio de Janeiro. It provides primary care services to residents of Manguinhos, which is a neighborhood with low household income. The clinic provides medical care free of charge to people who live in Manguinhos serving an average of 30,000 adults and 4,300 children ≤12 years old every month.

All children brought to the primary local health centre for immunisation, check-ups or emergency care were eligible to participate in the study, irrespective of whether or not they were symptomatic. At this time, the accompanying adult was invited to participate. Upon enrolment, a study nurse administered a questionnaire to the child's parent or guardian to collect data about the child's socio-demographic and clinical characteristics and medical history, as well as characteristics of the household. The questionnaire was completed on a tablet and took approximately 20 minutes. If the child was symptomatic an additional clinical questionnaire was completed by a paediatrician requiring approximately 30 more minutes. At the same time, we collected nasopharyngeal and serum samples from the child and any accompanying person. We also obtained the street address of the child's home, the parent or guardian's workplace (if different), and the telephone numbers of any relatives. This information was used to schedule home visits. Those who were approached but did not enrol in the study were either adults who stated that they did not have time to participate or children whose parent or legal guardian was not present at the time of the visit and therefore consent could not be obtained. Ethics approval is described below. A translation of the Informed Consent Form is included in the Supplementary Material.

Follow-up

In each household, we collect samples on Days 1 (first visit), 14 and 28, every three months during the first year and twice in the second year. All symptomatic participants were evaluated and

For peer review only - http://bmjopen.bmj.com/site/about/guidelines.xhtml

provided with medical care until symptoms resolved. The total follow-up time for each family was 24 months. At households with any symptomatic resident, an additional sample was collected on Day 7. Households in which no individual was examined during the entirety of a given epidemic wave were considered lost to follow-up after eight unsuccessful contact attempts by telephone and smartphone messaging apps. Individuals in each household who missed one or more study visits were excluded only if the study team was unable to contact the participant during the entire wave. Individuals with only one study visit were considered lost to follow-up.

Home visits were scheduled by phone and conducted by a team of field workers, including paediatricians and nurses. During these visits, study participation was offered to the child's household contacts, nasopharyngeal and serum samples were collected, and the GPS coordinates of the household were recorded. The study personnel completed a questionnaire for each contact who consented to participate consisting of clinical history, sociodemographic characteristics, and vaccination status. Visits lasted an average of 40 minutes. If time constraints prevented a participant from responding to all of the questions during the visit, the study team followed up with him or her by phone to complete the questionnaire.

Data collected

The structure of the participant database is shown in Figure 1. The information collected at baseline and each subsequent visit is summarised below:

- Sociodemographic characteristics: age, education, race, profession; residence: address, GPS coordinates, telephone, number of people living in the household, and number of rooms. With respect to race, the only legally accepted form of racial identification in Brazil is self-declaration. Data on race, schooling, and smoking were only collected for people 18 years of age (yoa) and older.

For peer review only - http://bmjopen.bmj.com/site/about/guidelines.xhtml

- Behavioural data: mask use, use of public transportation, visits to restaurants, bars, malls, movie theatres, barbeques, birthday parties, or other events where there was a crowd.
- Clinical manifestations in children: seizures, anosmia, dysgeusia, food refusal, fever, cough, nasal congestion, prostration, shortness of breath, abdominal pain, and diarrhoea.
- Adolescent/adult clinical variables: anosmia, dysgeusia, loss of appetite, fever, cough, rhinorrhoea, headache, prostration, shortness of breath, sore throat, abdominal pain, and diarrhoea.
- Vaccination status: number of doses, manufacturer, and vaccination date (after vaccination began in January 2021).
- Laboratory test results: SARS-CoV-2 RT-PCR test, SARS-CoV-2 lineage genomic test, and SARS-CoV-2 IgG anti-S antibody test of serum samples.

Nasopharyngeal swabs were tested by real time RT-PCR to amplify the E gene and the RdRp region of the Orf1ab gene of SARS-CoV-2 (Charité/Berlin, Germany). Cycle thresholds (CT) less than 40 were classified as positive. We assembled SARS-CoV-2 genomes from Illumina amplicons using the CLC Genomics Workbench v 20.0.4 (QIAGEN). Phylogenetic relationships among SARS-CoV-2 samples were determined using the Pango Lineages tool. SARS-CoV-2 serology (IgG) was performed using a Microparticle Chemiluminescent Immunoassay (CMIA), targeting the S gene (Abbott Laboratories, Abbott Park, IL, USA). All assays were performed according to the manufacturer's instructions. A study database was created with REDCap.[7 8]

FINDINGS TO DATE

In total, 79.5% of the individuals living in the same household as the child when approached consented to participate in the study. A total of 845 families were enrolled in the study: 34.2% in

For peer review only - http://bmjopen.bmj.com/site/about/guidelines.xhtml

the first epidemic wave, 16.4% in the Zeta wave, and 23.9% in the Gamma wave, 18.1% in the Delta wave, and 7.4% in the Omicron wave. This included 1152 children 12 and under, 758 of whom were retained in follow-up. As quarantine measures were relaxed, parents returned to working outside the home, which led to loss to follow-up of adults. This also resulted in loss to follow-up of children because children could not receive the study team in their homes without an adult present A total of 7,068 RT-PCR and 5,432 serology tests were performed.

We collected just one sample from 39.4% of the participants and therefore they were considered lost to follow-up. The racial composition of the lost to follow-up group and the retained in follow-up group were similar, as were years of schooling. However, the percentage of participants who were women differed between the groups. In the lost to follow-up group, 58% were women, whereas 62% of participants retained in follow-up were women. As the individuals eligible to participate in the study came from households with more women than men, it is unsurprising that women were more likely to be present during the study visits and be retained in follow-up than men. The retained and lost to follow-up groups also differed with respect to the proportion who were adults 18 years of age or older. Fifty-eight percent of the lost to follow-up group were 18 or older versus 48% in the retained in follow-up group.

Table 1. Demographic characteristics and schooling of individuals lost to follow-up versus those retained in the cohort.

Variables	Categories	Lost to follow-up	Retained in follow-up	*P*
Age (years)	0-12	404/1143 (35%)	758/1760 (43%)	<0.001
	13-17	74/1143 (7%)	153/1760 (9%)	
	18 or older	665/1143 (58%)	849/1760 (48%)	

For peer review only - http://bmjopen.bmj.com/site/about/guidelines.xhtml

Variables	Categories	Lost to follow-up	Retained in follow-up	*P*
Sex	Female	658/1143 (58%)	1095/1760 (62%)	0.012
	Male	485/1143 (42%)	665/1760 (38%)	
	Non white	433/665 (65%)	571/849 (67%)	
Race*	White	197/665 (30%)	250/849 (30%)	0.7
	Missing	35/665 (5%)	28/849 (3%)	
Completed primary school*	Yes	473/665 (71%)	651/849 (77%)	
	No	138/665 (21%)	159/849 (19%)	0.2
	Missing	54/665 (8%)	39/849 (4%)	

*Race and schooling were only collected for adults.

The average number of participants recruited per week was 27.1. The period of follow-up was 24 months. As enrolment began in May 2020 and we followed participants through May 2022, some participants had completed their schedule of visits before the end of the study (Figure 2). Those who were recruited recently have not completed all of their follow-up visits. Currently, 67.5% of the participants who were enrolled at any time are still being followed. The mean follow-up time was 235 days for participants ≥ 13 years old and 261 days for those ≤ 12 years old. The mean follow-up time was greater for women ≥ 13 years old (247 days) than for men in the same age group (208 days).

Our cohort can be analysed at the household scale or at the individual scale. At the household scale, the sociodemographic composition of the domiciles was similar throughout the study. The median number of residents per household was four. The median household density, defined as the number of persons per room, was 0.95. The cohort can also be analysed at the individual

For peer review only - http://bmjopen.bmj.com/site/about/guidelines.xhtml

scale, without considering the household to which each individual belonged. At the individual scale, most sociodemographic characteristics were similar across the entire period, except for the Omicron wave, in which there were fewer participants (Table 2). The majority of participants were women (62.3%), reflecting not only the recruitment strategy, as children are usually accompanied by their mothers or grandmothers, but also the caregiver at home. The median age of the participants was 14-19 during the first four waves of the participants but decreased to nine years of age during the Omicron wave. Across all five waves of the pandemic, the interquartile range of participant ages was approximately five to thirty-five years old. The self-declared race of 70% of the adult participants was non-white. Across all recruitment periods, 45-54% of the participants were adults, and 36-52% were children ≤ 12 years old (Table 2). More than 80% of the adult participants had completed primary school. The prevalence of smoking among adults was 9%.

Table 2. Demographic characteristics of the cohort participants by epidemic wave.

Variable	Overall	B.1.1.33	Zeta	Gamma	Delta	Omicron
	**Median (IQR)**	**Median (IQR)**	**Median (IQR)**	**Median (IQR)**	**Median (IQR)**	**Median (IQR)**
Days of follow-up	222 (105-365)	317 (196-522)	265 (147-426)	162 (68-256)	137 (49-197)	27 (14-93)
Age (years)	16 (7-35)	14 (5-36)	19 (7-35)	19 (8-38)	15 (8-32)	8 (4-24)
	**N (%)**	**N (%)**	**N (%)**	**N (%)**	**N (%)**	**N (%)**

For peer review only - http://bmjopen.bmj.com/site/about/guidelines.xhtml

Sex	Male	665/1760 (38%)	239/623 (38%)	131/343 (38%)	174/464 (37%)	106/287 (37%)	15/43 (35%)
	Female	1095/1760 (62%)	384/623 (62%)	212/343 (62%)	290/464 (63%)	181/287 (63%)	28/43 (65%)
Age groups	0-12	758/1760 (43%)	289/623 (46%)	138/343 (40%)	173/464 (37%)	129/287 (45%)	29/43 (67%)
	13-17	153/1760 (9%)	46/623 (8%)	28/343 (8%)	51/464 (11%)	28/287 (10%)	0
	>= 18	849/1760 (48%)	288/623 (46%)	177/343 (52%)	240/464 (52%)	130/287 (45%)	14/43 (33%)
Race*	White	250/849 (29%)	81/288 (28%)	57/177 (32%)	71/240 (30%)	36/130 (28%)	5/14 (36%)
	Non white	571/849 (67%)	204/288 (71%)	113/177 (64%)	154/240 (64%)	91/130 (70%)	9/14 (64%)
	Missing	28/849 (4%)	3/288 (1%)	7/177 (4%)	15/240 (6%)	3/130 (2%)	0
Completed primary school*	Yes	651/849 (77%)	219/288 (76%)	138/177 (78%)	179/240 (75%)	105/130 (81%)	10/14 (71%)
	No	159/849 (19%)	65/288 (23%)	28/177 (16%)	43/240 (18%)	19/130 (15%)	4/14 (29%)
	Missing	39/849 (4%)	4/288 (1%)	11/177 (6%)	18/240 (7%)	6/130 (4%)	0

For peer review only - http://bmjopen.bmj.com/site/about/guidelines.xhtml

Smoking*	Yes	73/849 (9%)	28/288 (9%)	10/177 (6%)	11/240 (5%)	16/130 (13%)	8/14 (57%)
	No	752/849 (89%)	258/288 (90%)	160/177 (90%)	217/240 (90%)	111/130 (85%)	6/14 (43%)
	Missing	24/849 (2%)	3/288 (1%)	7/177 (4%)	11/240 (5%)	3/130 (2%)	0

*Race, schooling, and smoking were only collected for adults.

The seroprevalence of SARS-CoV-2 antibodies indicative of past exposure to the virus or a vaccine was approximately 20% from May 2020 to June 2021. Seroprevalence subsequently increased precipitously, likely reflecting the rollout of SARS-CoV-2 vaccination (Figure 3). Complete vaccination (two doses of BNT162b2, AZD1222, CoronaVac (Sinovac), or one dose of Ad26.COV2.S) increased with age and was over 80% for all but the youngest age groups (Figure 4).

The first published report from this cohort provided evidence that children were not the principal source of SARS-CoV-2 infections in their households during the first wave of the pandemic but more frequently acquired the virus from adolescent and adult household contacts.[9] In a subsequent study, we estimated the incidence of SARS-CoV-2 in the cohort during the B.1.1.33, Zeta, Gamma, and Delta waves.[6] Household crowding was significantly associated with SARS-CoV-2 incidence only during the first wave.[6] Through the Delta wave, the incidence among children was lower than that of older participants. Subsequently, vaccination of the elderly reached 90% and incidence among children was higher.

For peer review only - http://bmjopen.bmj.com/site/about/guidelines.xhtml

For peer review only

PATIENT AND PUBLIC INVOLVEMENT

The study personnel include residents of Manguinhos who were first hired during our previous cohort studies.[9-13] These professionals are considered trustworthy by members of the community and permitted to move through the area freely. This enables them to find households that would be extremely difficult for someone unfamiliar with the community to locate. In addition, all study participants were given the opportunity to provide feedback about the design of the study during the first home visit.

STRENGTHS AND LIMITATIONS OF THIS STUDY

Our study data are unique because this is a community that is not easily accessible to researchers due to high levels of poverty and violence. Nevertheless, it is emblematic of low-resource communities in other cities in Latin America. The study's high rates of participant retention and visit completion are among its principal strengths. Furthermore, since we recruited asymptomatic and symptomatic participants, we were able to estimate the overall incidence of SARS-CoV-2 infection in this population, not restricted to individuals with obvious clinical signs of COVID-19. Our study team included personnel from different disciplines including epidemiology, medicine, virology, immunology, and statistics. We believe that this strengthened the research because we were able to examine the ramifications of the COVID-19 pandemic for this community from a number of different angles. As the field workers were people living in the community, they were able to easily establish a link with participants. Finally, since the cohort has been followed continuously for two years, we have been able to gather data about successive variants that have swept through the population. The main limitation is that in some cases it was

For peer review only - http://bmjopen.bmj.com/site/about/guidelines.xhtml

not possible to carry out visits in accordance with the timeline in the study protocol. The principal reasons for missed visits were armed conflicts within the community or work obligations outside the community. We may have failed to detect acute SARS-CoV-2 infections that occurred at the time of missed visits.

COLLABORATION

While the data at the individual level are not openly available to preserve the anonymity of the study participants, researchers who are interested in potential collaboration should contact the corresponding author. The study steering committee will evaluate data requests in accordance with best practices in cohort studies to ensure that data sharing would preserve confidentiality and be compatible with the terms of the Informed Consent Form used in this study.

FUTURE PLANS

The biobank of cryopreserved peripheral blood mononuclear cells (PBMC) and serum from the cohort participants will allow future studies of cellular and humoral immune responses to locally circulating SARS-CoV-2 variants, which is relevant for the design of future vaccines, antivirals, and monoclonal antibodies. We will also analyse the immune profile of participants who experienced breakthrough infections following vaccination. Of course, in order for novel SARS-CoV-2 variants and other emerging pathogens to be controlled, it will be necessary to reduce transmission. To this end, future work will also involve publicising virus control and prevention practices. We thus plan to create a short ‘edutainment’ style video to disseminate our results and encourage SARS-CoV-2 immunisation. The target audience will be children and families. As

For peer review only - http://bmjopen.bmj.com/site/about/guidelines.xhtml

part of these efforts, we will work closely with media partners, policymakers and the cohort participants to develop effective outreach strategies. We believe such activities might help bring about better compliance with COVID-19 control measures not only in this community, but also at the regional and global levels.

COMPETING INTERESTS

All authors declare no competing interests.

ACKNOWLEDGMENTS

Thanks are due to the families in Manguinhos who kindly agreed to participate in the study. This research would not have been possible without the support of Carlos Alberto de Moraes Costa, Iris Maria da Silva Lordello, and the staff of the Germano Sinval Faria Health Centre, the Oswaldo Cruz Foundation's COVID-19 Diagnosis Support Unit, Vice-Presidency of Health Production and Innovation, and LabiExames.

FUNDING

Ministério da Ciência, Tecnologia e Inovação Conselho Nacional de Desenvolvimento Científico e Tecnológico 3041101/2017-6, 307450/2021-0, 307282/2017-1, Fundação Carlos Chagas Filho de Amparo à Pesquisa do Estado do Rio de Janeiro E-26/201.356/2014 E-26/202.862/2018, E-26/211.565/2019, E-26/210.149/2020; UK Research and Innovation MR/V033530/1.

For peer review only - http://bmjopen.bmj.com/site/about/guidelines.xhtml

ETHICS APPROVAL

The study was approved by the Brazilian National Ethics Committee (CONEP) under protocol number 30639420.0.0000. 5262. All participants gave their informed consent to participate in the study.

CONTRIBUTORS

Patricia Brasil: conceptualization, methodology, writing - original draft, writing - review and editing, project administration, and supervision. Luana Damasceno: data curation and methodology. Trevon Fuller: methodology, writing - original draft, writing - review and editing. Leonardo S. Bastos: visualization and data curation. Oswaldo G. Cruz: methodology, visualization and data curation. Fernando Medeiros: software and data curation. Guilherme Calvet: data curation. Paola Cristina Resende: resources. Jimmy Whitworth: funding acquisition and methodology. Chris Smith: funding acquisition and methodology. Marilda Siqueira: resources, Marilia Carvalho: conceptualization, methodology, writing - original draft, writing - review and editing. All authors provided intellectual contributions to the manuscript and approved the final manuscript.

For peer review only

REFERENCES

1. World Health Organization. News Release: 14.9 million excess deaths associated with the COVID-19 pandemic in 2020 and 2021. Geneva: Switzerland. Geneva: WHO 2022.

For peer review only - http://bmjopen.bmj.com/site/about/guidelines.xhtml

2. Brazilian Ministry of Health. Brazilian Coronavirus Dashboard. Available at: https://covid.saude.gov.br/ Accessed 21 October 2022. Brasília: Brazilian Ministry of Health 2022.

3. World Health Organization. Coronavirus (COVID-19) Dashboard. Available at: https://covid19.who.int/. Accessed 21 October 2022. Geneva: WHO 2022.

4. Madewell ZJ, Yang Y, Longini IM, Jr, et al. Factors Associated With Household Transmission of SARS-CoV-2: An Updated Systematic Review and Meta-analysis. *JAMA Network Open* 2021;4(8):e2122240-e40. doi: 10.1001/jamanetworkopen.2021.22240

5. Resende PC, Delatorre E, Gräf T, et al. Evolutionary Dynamics and Dissemination Pattern of the SARS-CoV-2 Lineage B.1.1.33 During the Early Pandemic Phase in Brazil. *Frontiers in Microbiology* 2021;11 doi: 10.3389/fmicb.2020.615280

6. Carvalho MS, Bastos LS, Fuller T, et al. Incidence of SARS-CoV-2 over four epidemic waves in a low-resource community in Rio de Janeiro, Brazil: A prospective cohort study. *The Lancet Regional Health – Americas* 2022;12 doi: 10.1016/j.lana.2022.100283

7. Harris PA, Taylor R, Minor BL, et al. The REDCap consortium: Building an international community of software platform partners. *Journal of biomedical informatics* 2019;95:103208. doi: 10.1016/j.jbi.2019.103208 [published Online First: 2019/05/13]

8. Harris PA, Taylor R, Thielke R, et al. Research electronic data capture (REDCap)--a metadata-driven methodology and workflow process for providing translational research informatics support. *Journal of biomedical informatics* 2009;42(2):377-81. doi: 10.1016/j.jbi.2008.08.010 [published Online First: 2008/10/22]

For peer review only - http://bmjopen.bmj.com/site/about/guidelines.xhtml

9. Lugon P, Fuller T, Damasceno L, et al. SARS-CoV-2 infection dynamics in children and household contacts in a slum in Rio de Janeiro. *Pediatrics* 2021;148(1) doi: 10.1542/peds.2021-050182 [published Online First: 2021/04/18]

10. Pedro RS, Carvalho MS, Girianelli VR, et al. A populational-based birth cohort study in a low-income urban area in Rio de Janeiro, Brazil: implementation and description of the characteristics of the study. *Cadernos de Saúde Pública* 2019;35

11. Aguiar DF, de Barros ENC, Ribeiro GS, et al. A prospective, multicentre, cohort study to assess the incidence of dengue illness in households from selected communities in Brazil (2014-2018). *International journal of infectious diseases* 2021;108:443-53. doi: 10.1016/j.ijid.2021.04.062 [published Online First: 2021/04/25]

12. Damasceno L, Terzian ACB, Fuller T, et al. Why did ZIKV perinatal outcomes differ in distinct regions of Brazil? An exploratory study of two cohorts. *Viruses* 2021;13(5):736.

13. Brasil P, Pereira JP, Moreira ME, et al. Zika virus infection in pregnant women in Rio de Janeiro. *New England Journal of Medicine* 2016;375(24):2321-34. doi: 10.1056/NEJMoa1602412

For peer review only

Figure Captions

Figure 1. Cohort database structure.

Figure 2. Recruitment and retention by SARS-CoV-2 epidemic wave.

Figure 3. SARS-CoV-2 anti-S seroprevalence by month of sample collection.

Figure 4. SARS-CoV-2 vaccine coverage by age group.

For peer review only - http://bmjopen.bmj.com/site/about/guidelines.xhtml

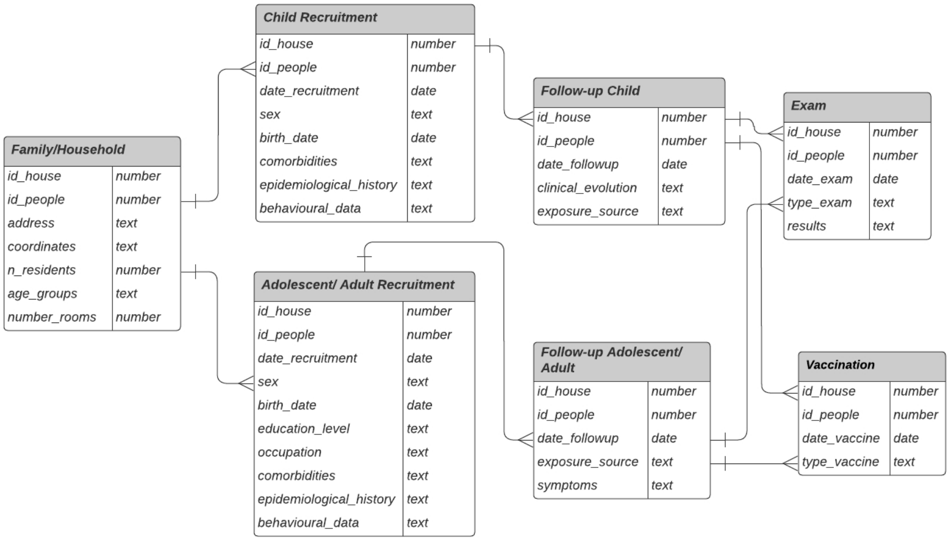

Figure 1. Cohort database structure.

548x324mm (63 x 63 DPI)

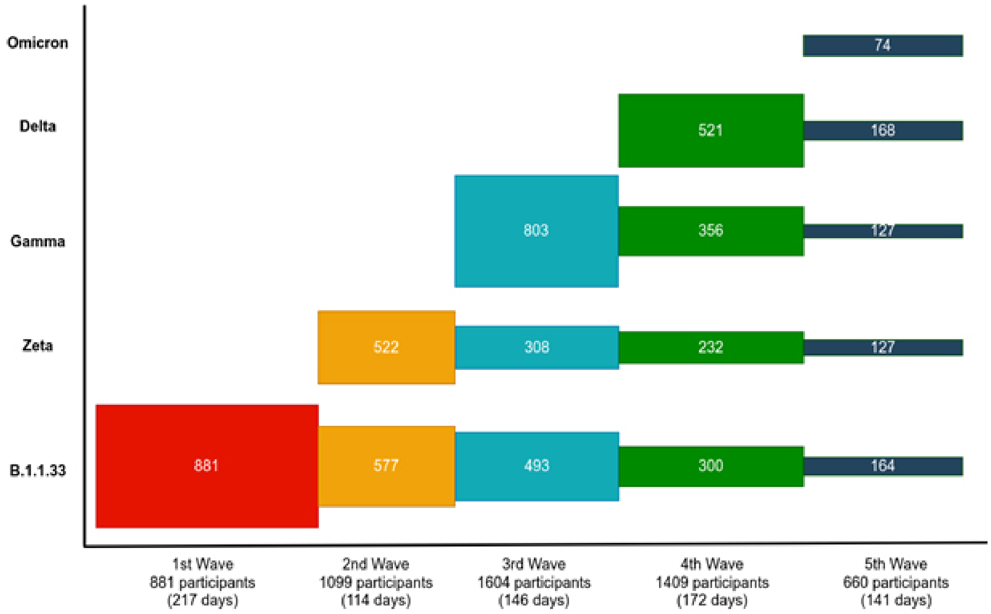

Figure 2. Recruitment and retention by SARS-CoV-2 epidemic wave.

220x140mm (72 x 72 DPI)

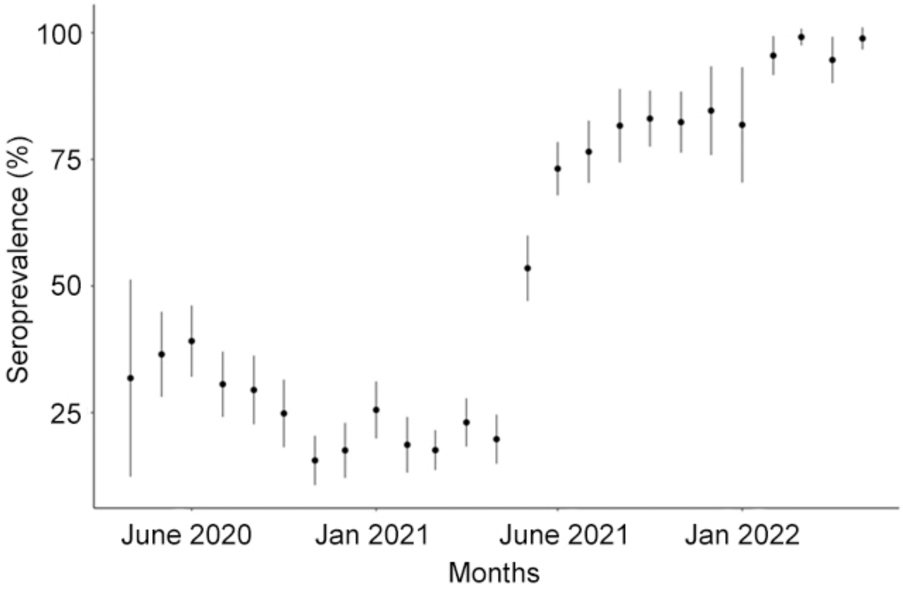

Figure 3. SARS-CoV-2 anti-S seroprevalence by month of sample collection.

643x392mm (28 x 28 DPI)

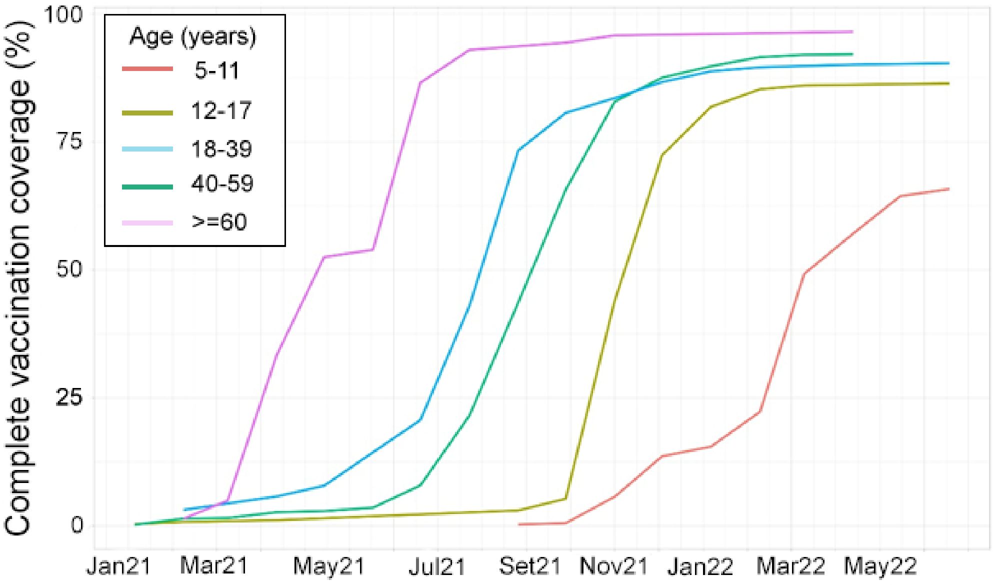

Figure 4. SARS-CoV-2 vaccine coverage by age group.

552x392mm (28 x 28 DPI)

Supplementary Material

Informed Consent Form

Household Transmission and Immunity to SARS-CoV-2 among Paediatric Clients of a Primary Care Center in a Low-resource Community in Rio de Janeiro.

You are being invited to participate in a study that will investigate the coronavirus, hereafter SARS-CoV-2, among people without symptoms because you may have been exposed to the virus or because your SARS-CoV-2 test was positive.

Since December 2019, SARS-CoV-2 has spread rapidly in China and around the world. As of November 2020, 47 million cases have been reported in 189 countries. During this period, a total of 5.6 million cases were reported in Brazil.

The aim of this study is to investigate the clinical progression of SARS-CoV-2 in persons with and without symptoms. In connection with this, the study will assess whether your body is producing antibodies and other substances in response to the virus. We will also study whether people who live or work in your home become infected.

If you agree to participate in the study we will complete a form with information about your symptoms, related medical conditions, and any medications that you take. With your permission, we will collect 4 mL of your blood, which is approximately the same volume as a soup spoon. The blood will be collected via a venal puncture with a disposable needle by a nurse or a trained lab technician. The blood sample will be used to carry out lab exams to detect antibodies or markers of inflammation caused by SARS-CoV-2 infection. We will also collect nasal and throat secretions using three long, thin, and sterile cotton swabs (Q-tips). A swab will be placed in your mouth and

For peer review only - http://bmjopen.bmj.com/site/about/guidelines.xhtml

moved in the direction of your tonsils. Another swab will be placed in each nostril and moved in the direction of your nasopharynx, which is approximately 6-8 cm deep within the nasal cavity. We will also collect a fecal sample via a Q-tip inserted into your anus to a depth of approximately 1 cm. You can collect the fecal sample yourself following instructions that we will give you. We will search for SARS-CoV-2 in these samples and attempt to determine its characteristics.

If your test for the coronavirus is negative, we will stay in touch with you to follow the progression of your symptoms. If your test is positive, you will be accompanied by the study for about six weeks. This will involve doing the exams mentioned above about once a week, for a total of 3-4 sample collections. The purpose of the exams will be to see for how long you will have the virus and to detect the appearance of antibodies. If you need to be hospitalized, these tests will be done every 48 hours, on the day when you are released, and six weeks later. You can participate in this study via phone/WhatsApp, social media (Facebook), video calls, or home visits.

As part of this study, we will remove your identifiable, confidential information from the database then share it with foreign collaborators for future analyses.

Your blood sample will be analyzed and stored at the Laboratory for Clinical Research on Acute Febrile Illnesses. Your nasal and throat secretions and feces will be stored at the Laboratory for Respiratory Viruses and Measles. Both labs are located at 4365 Avenida Brasil on the Manguinhos Campus of Fiocruz in Rio de Janeiro. The researchers conducting this study will be responsible for the storage of your samples. You can withdraw consent for the storage of your biological samples whenever you wish. You should let us know in writing that you wish to withdraw, and sign and date your request. Your samples will then be destroyed without affecting your medical care.

For peer review only - http://bmjopen.bmj.com/site/about/guidelines.xhtml

We request your authorization to store your samples for up to ten years. Future studies with the samples will be important for the development of new diagnostics, treatments, and vaccines. Such research will only be carried out following approval by the Brazilian National Research Ethics Committee and/or the Ethics Committee of the National Institute of Infectious Diseases at Fiocruz. It may be necessary for us to seek your free and informed written consent before carrying out such future research. If you do not agree to the use of your biological samples in future studies, your samples will be thrown away at the end of this study.

The greatest risks associated with your participation in this study are those related to: the blood draw, which can cause pain or redness at the site of the puncture; the collection of the throat or nasal swabs, which do not typically cause pain, but may cause light discomfort, cough, nausea, eye watering, the urge to sneeze, or light bleeding. These risks will be minimized because the procedures will be carried out by trained professionals. A nurse will give you one-on-one instructions so that you will not feel uncomfortable when collecting the fecal sample.

Another risk associated with this study is that there could be a loss of confidentiality. However, during the analysis of the data, we will replace your name with a code. Confidential information will not be transmitted over the internet. Your privacy will be guaranteed by the fact that your personal data will be de-identified and the results will be presented in an aggregated format in vehicles of scientific communication.

The benefits that this study may produce are information and guidelines that can improve the detection of SARS-CoV-2 by identifying the age groups that have the greatest risk of

For peer review only - http://bmjopen.bmj.com/site/about/guidelines.xhtml

contracting the virus. This can contribute to the implementation of public health policies and improve scientific knowledge.

Your participation in the study is entirely voluntary and you can refuse to take part or stop at any time without giving us a reason. Your withdrawal will not affect your access to medical care. If you decide not to participate in the study, we will offer you standard medical care including recommendations about public clinics and hospitals where you can receive care later if necessary.

If your SARS-CoV-2 test is positive, you will received all necessary care and medical advice. We guarantee your right to complete medical care free of charge for as long as necessary should any harm befall you due to your participation in this study.

In accordance with Brazilian law, you have the right to seek damages if you are harmed in the study.

At any time during or after the study you have the right to ask the researcher for information regarding your participation in the study. This request can be made via the contact information listed on this form. We guarantee that you will have access to the results of any medical test carried out during the study whenever you or your legal representative requests it. You will also have access to the overall results obtained at the end of the project.

You will not receive financial compensation for participating in this study. All costs associated with the study will be covered by the principal investigator. In other words, you will incur no expense for study procedures or exams.

For peer review only - http://bmjopen.bmj.com/site/about/guidelines.xhtml

We have printed two copies of this form, one of which will be made available to you and the other of which will be kept by the researcher. Every page should be initialed by you and either by the researcher in charge or by someone to whom she has delegated responsibility for signing.

For more information, please contact the Principal Investigator of the study: Dr. Patrícia Brasil. Av. Brasil 4365, Instituto Nacional de Infectologia Evandro Chagas – Fiocruz, sala 109, subsolo do hospital. Telephones: 3865-9115 or 3965-9110

Should you have any questions about the ethical conduct of the study, please contact the Committee for Ethics in Research at the National Institute of Infectious Diseases at the Oswaldo Cruz Foundation. The Ethics Committee is a body whose purpose is to defend the interests of research study participants preserving their dignity and personhood while also contributing to the advancement of research that respects ethical norms. Thus, the Committee's role is to evaluate and monitor the conduct of the research project in such a way as to respect ethical principles that respect human rights, individual dignity, autonomy, benevolence, confidentiality, and privacy.

Comitê de Ética em Pesquisa Instituto Nacional de Infectologia Evandro Chagas (INI) Manguinhos - Rio de Janeiro - RJ Tel: 3865-9595 (Business hours: Monday-Friday 9am to 5 pm)

If you wish, you also may contact the Brazilian National Committee for Research Ethics (Conep): Tel: (61) 3315-5878 / (61) 3315-5879. E-Mail: conep@saude.gov.br

For peer review only - http://bmjopen.bmj.com/site/about/guidelines.xhtml

I understand all of these instructions and therefore authorize the professionals of Fiocruz to use my blood samples, nasal and throat secretions, and feces while preserving the confidentiality of the results, which will be made available to me individually. The results may also be reported only to public health institutions (municipal and state health departments and the Ministry of Health), and can be published in scientific vehicles of communication.

( ) I also authorize the storage and use of my biological material collected during this study for future studies.

( ) I do not authorize the storage of my samples for future studies.

Date: _____/_____/__________

Name of the participant:

____

Participant's signature:

____

Signature of the researcher responsible:

____

For peer review only

For peer review only - http://bmjopen.bmj.com/site/about/guidelines.xhtml

STROBE 2007 (v4) Statement—Checklist of items that should be included in reports of *cohort studies*

Section/Topic	Item #	Recommendation	Reported on page #
**Title and abstract**	1	(*a*) Indicate the study's design with a commonly used term in the title or the abstract	1
		(*b*) Provide in the abstract an informative and balanced summary of what was done and what was found	2
**Introduction**			
Background/rationale	2	Explain the scientific background and rationale for the investigation being reported	3
Objectives	3	State specific objectives, including any prespecified hypotheses	4
**Methods**			
Study design	4	Present key elements of study design early in the paper	4
Setting	5	Describe the setting, locations, and relevant dates, including periods of recruitment, exposure, follow-up, and data collection	4
Participants	6	(*a*) Give the eligibility criteria, and the sources and methods of selection of participants. Describe methods of follow-up	5
		(*b*) For matched studies, give matching criteria and number of exposed and unexposed	NA
Variables	7	Clearly define all outcomes, exposures, predictors, potential confounders, and effect modifiers. Give diagnostic criteria, if applicable	7
Data sources/ measurement	8*	For each variable of interest, give sources of data and details of methods of assessment (measurement). Describe comparability of assessment methods if there is more than one group	6
Bias	9	Describe any efforts to address potential sources of bias	5
Study size	10	Explain how the study size was arrived at	8
Quantitative variables	11	Explain how quantitative variables were handled in the analyses. If applicable, describe which groupings were chosen and why	6
Statistical methods	12	(*a*) Describe all statistical methods, including those used to control for confounding	NA
		(*b*) Describe any methods used to examine subgroups and interactions	NA
		(*c*) Explain how missing data were addressed	NA
		(*d*) If applicable, explain how loss to follow-up was addressed	5
		(*e*) Describe any sensitivity analyses	NA
**Results**			

For peer review only

Participants	13*	(a) Report numbers of individuals at each stage of study—eg numbers potentially eligible, examined for eligibility, confirmed eligible, included in the study, completing follow-up, and analysed	7
		(b) Give reasons for non-participation at each stage	8
		(c) Consider use of a flow diagram	Figure 1
Descriptive data	14*	(a) Give characteristics of study participants (eg demographic, clinical, social) and information on exposures and potential confounders	9
		(b) Indicate number of participants with missing data for each variable of interest	NA
		(c) Summarise follow-up time (eg, average and total amount)	8
Outcome data	15*	Report numbers of outcome events or summary measures over time	9
Main results	16	(*a*) Give unadjusted estimates and, if applicable, confounder-adjusted estimates and their precision (eg, 95% confidence interval). Make clear which confounders were adjusted for and why they were included	NA
		(*b*) Report category boundaries when continuous variables were categorized	5
		(*c*) If relevant, consider translating estimates of relative risk into absolute risk for a meaningful time period	NA
Other analyses	17	Report other analyses done—eg analyses of subgroups and interactions, and sensitivity analyses	NA
**Discussion**			
Key results	18	Summarise key results with reference to study objectives	10
**Limitations**			
Interpretation	20	Give a cautious overall interpretation of results considering objectives, limitations, multiplicity of analyses, results from similar studies, and other relevant evidence	11
Generalisability	21	Discuss the generalisability (external validity) of the study results	10
**Other information**			
Funding	22	Give the source of funding and the role of the funders for the present study and, if applicable, for the original study on which the present article is based	13

*Give information separately for cases and controls in case-control studies and, if applicable, for exposed and unexposed groups in cohort and cross-sectional studies.

Note: An Explanation and Elaboration article discusses each checklist item and gives methodological background and published examples of transparent reporting. The STROBE checklist is best used in conjunction with this article (freely available on the Web sites of PLoS Medicine at http://www.plosmedicine.org/, Annals of Internal Medicine at http://www.annals.org/, and Epidemiology at http://www.epidem.com/). Information on the STROBE Initiative is available at www.strobe-statement.org.

For peer review only
